# Economic evaluation of the effect of needle and syringe programs on skin, soft tissue, and vascular infections in people who inject drugs: a microsimulation modelling approach

**DOI:** 10.1186/s12954-024-01037-3

**Published:** 2024-06-28

**Authors:** Jihoon Lim, W. Alton Russell, Mariam El-Sheikh, David L. Buckeridge, Dimitra Panagiotoglou

**Affiliations:** Department of Epidemiology, Biostatistics, and Occupational Health, McGill University, 2001 McGill College Avenue, Suite 1200, Montreal, QC H3A 1G1 Canada

**Keywords:** Economic modelling, Microsimulation, Recurrent-event survival analysis, Needle and syringe program, Skin, Soft tissue and vascular infections

## Abstract

**Background:**

Needle and syringe programs (NSP) are effective harm-reduction strategies against HIV and hepatitis C. Although skin, soft tissue, and vascular infections (SSTVI) are the most common morbidities in people who inject drugs (PWID), the extent to which NSP are clinically and cost-effective in relation to SSTVI in PWID remains unclear. The objective of this study was to model the clinical- and cost-effectiveness of NSP with respect to treatment of SSTVI in PWID.

**Methods:**

We performed a model-based, economic evaluation comparing a scenario with NSP to a scenario without NSP. We developed a microsimulation model to generate two cohorts of 100,000 individuals corresponding to each NSP scenario and estimated quality-adjusted life-years (QALY) and cost (in 2022 Canadian dollars) over a 5-year time horizon (1.5% per annum for costs and outcomes). To assess the clinical effectiveness of NSP, we conducted survival analysis that accounted for the recurrent use of health care services for treating SSTVI and SSTVI mortality in the presence of competing risks.

**Results:**

The incremental cost-effectiveness ratio associated with NSP was $70,278 per QALY, with incremental cost and QALY gains corresponding to $1207 and 0.017 QALY, respectively. Under the scenario with NSP, there were 788 fewer SSTVI deaths per 100,000 PWID, corresponding to 24% lower relative hazard of mortality from SSTVI (hazard ratio [HR] = 0.76; 95% confidence interval [CI] = 0.72–0.80). Health service utilization over the 5-year period remained lower under the scenario with NSP (outpatient: 66,511 vs. 86,879; emergency department: 9920 vs. 12,922; inpatient: 4282 vs. 5596). Relatedly, having NSP was associated with a modest reduction in the relative hazard of recurrent outpatient visits (HR = 0.96; 95% CI = 0.95–0.97) for purulent SSTVI as well as outpatient (HR = 0.88; 95% CI = 0.87–0.88) and emergency department visits (HR = 0.98; 95% CI = 0.97–0.99) for non-purulent SSTVI.

**Conclusions:**

Both the individuals and the healthcare system benefit from NSP through lower risk of SSTVI mortality and prevention of recurrent outpatient and emergency department visits to treat SSTVI. The microsimulation framework provides insights into clinical and economic implications of NSP, which can serve as valuable evidence that can aid decision-making in expansion of NSP services.

**Supplementary Information:**

The online version contains supplementary material available at 10.1186/s12954-024-01037-3.

## Introduction

Injection drug use and associated high-risk injecting behaviours (e.g., needle or syringe sharing) are a major public health issue, as they increase the risk of overdose [1] and HIV and hepatitis C virus (HCV) infection [2–4]. However, harms associated with injection drug use extend beyond overdose and blood borne infections.

Among people who inject drugs (PWID), skin, soft tissue, and vascular infections (SSTVI) at drug injection sites are the leading cause of emergency department (ED) visits and hospitalizations, globally [5–12]. SSTVI are bacterial infections of the skin and subcutaneous soft tissues, which can lead to inflammatory response (e.g., pain and swelling) as well as formation of lesions and bullae [13]. Some cases of SSTVI may manifest as abscess or cellulitis, which can be treated with antibiotics or incision and drainage procedures [14]. However, without timely treatment, SSTVI can progress into necrotizing infections or sepsis [15]. Sharing and reusing of injection equipment, frequency of injections, and years of injection drug use are all known to increase the risk of SSTVI [16–19].

In Canada, a confluence of factors may be contributing to increasing SSTVI morbidity among PWID [20, 21]. In recent years, changes in consumption patterns owing to the introduction of more powerful but shorter acting synthetic opioids such as fentanyl in the unregulated market means the frequency of injection drug use may be on the rise [22–24]. Meanwhile, because PWID limit their use of health services owing to past experiences of mistreatment and/or stigma [25], treatment for SSTVI is often delayed [26], leading to more serious morbidity and costly hospitalizations [27].

One of the first harm reduction strategies employed was needle and syringe programs (NSP), where PWID are provided with sterile hypodermic needles and other equipment at low to no cost. In Montreal, Canada, NSP started as a grassroots community initiative under CACTUS-Montreal (*Centre d’Action Communautaire auprès des Toxicomanes Utilisateurs de Seringues*) in 1989 [28], with the aim of preventing HIV transmission [29]. An early study of Quebec’s NSP found that there was an 11% decline in the needle sharing rate (31% to 20%) within two years of beginning operation [30]. More recent studies on NSP reveal that these programs are associated with significant reductions in HIV and HCV transmission among PWID [31, 32]. In addition, NSP may help mitigate SSTVI, as they reduce needle sharing and reuse of injection equipment while contributing to injection cessation [33–38]. The reduction in risky injection behaviour and promotion of injection cessation are achieved through outreach programs that accompany NSP, which include education on safe and hygienic injection practices and needle disinfection [36].

Despite evidence of lower injection risk behaviour associated with NSP, Canadian modelling studies of harm reduction interventions have focused on supervised consumption sites and their role in prevention of HIV and HCV transmission [39–42]. A recent study found cost-effectiveness of NSP in relation to mitigating fatal overdose events [43], but the cost-effectiveness of NSP relative to SSTVI remains uncertain. Additionally, the impact of NSP on the public health burden of SSTVI and the health service utilization patterns for treating SSTVI among PWID is unclear. By addressing these knowledge gaps, we will be able to demonstrate the economic value of NSP as well as whether there are additional benefits of NSP beyond prevention of HIV and HCV. Therefore, in this study, we aim to model the cost-effectiveness of NSP versus not having NSP with a focus on the treatment of SSTVI among PWID.

## Methods

### Model type and health states

**Fig. 1:**
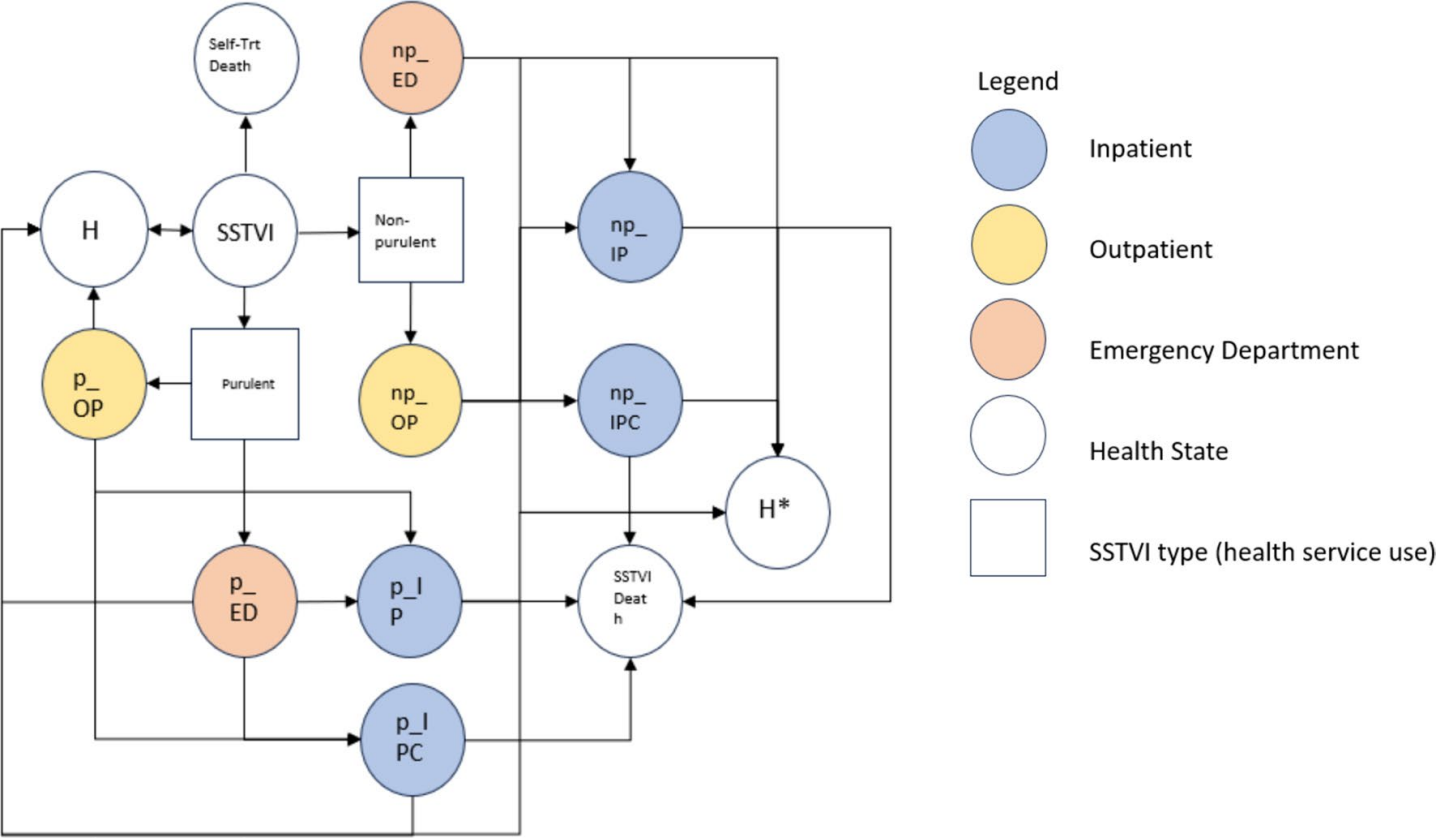
Model schematic for progression of SSTVI in PWID. Keys: H = healthy; np = non-purulent; p = purulent; OP = outpatient; ED = emergency department; IP = inpatient; IPC = inpatient complications; SSTVI = skin, soft tissue, and vascular infections; Trt = treatment. Note: H and H* indicate the same health state with the same health state utilities. For ease of visualization, we created the H* state in the diagram

We constructed a 13-state transition microsimulation model to capture patterns of progression of SSTVI and the corresponding health service utilization in a population of PWID (Fig. 1). We chose this individual-based, state-transition model due to its ability to model each individual’s unique clinical pathways, incorporate individual’s history in the occurrence of future events, and handle a relatively large number of health states [44, 45]. To model the transmission of SSTVI, we chose a microsimulation framework as opposed to an agent-based framework, as the proportion of PWID who shared needles with other individuals was low (15%) [46]. The health states were based on disease progression, treatment guidelines, and health service utilization patterns for treating SSTVI (see Supplementary Materials for the list of health states) [14, 47]. We set the time horizon for this model as 5 years with the cycle length of 1 week. We chose this specific cycle length because clinical practice guidelines explicitly state that the duration of antibiotic treatment be 5–7 days [14, 47, 48], and the 7-day period is often used to identify separate episodes of SSTVI or identify complications from an existing episode [9]. Details of the model assumptions are described in Supplementary Materials.

### Data sources

To derive the parameters for our model, we used six administrative health databases provided by the Government of Québec (see Supplementary Materials for more detail; Research file publication date: 2009–2019; Data use approval date: 20 September 2021).^1^ Further information regarding these datasets can be found by visiting the *Institut de la Statistique du Québec* (ISQ) webpage at: https://statistique.quebec.ca/services-recherche/donnees/administratives [49]. All inferences, opinions, and conclusions drawn in this publication are those of the authors, and the Government of Québec is not responsible for the compilations or the interpretation of the results produced using the research files.^2^

To identify values for model parameters that influenced the characteristics of injection practices among PWID, we used the results from surveillance of PWID conducted by Quebec Provincial Public Health Institute (INSPQ) [46]. Finally, we retrieved the health state utility weight associated with varying degrees of severity of SSTVI (e.g., outpatient versus inpatient) from the published literature [50–53].

### Model structure

Figure 1 illustrates the model schematic of how PWID with SSTVI treat their infection. The model schematic and health states informing the model were adopted from the Infectious Disease Society of America’s (IDSA) “Practice Guidelines for the Diagnosis and Management of Skin and Soft Tissue Infections” [14] and the University of California San Francisco (UCSF) Infectious Disease Management Program’s “Guideline for the Management of Suspected Skin and Soft Tissue Infections in Adults” [47]. The model reflects how individuals transition through states of self-treatment or care within the healthcare system until the infection is resolved, they die, or reach the end of the time horizon.

Our model includes two types of SSTVI: (1) purulent and (2) non-purulent (International Classification of Diseases [ICD]-9/10 codes in Supplementary Materials). Purulent SSTVI include abscess, furuncle, carbuncle, and folliculitis. Non-purulent SSTVI include cellulitis, erysipelas, and necrotizing fasciitis. To account for deaths from other causes (e.g., overdose, injury, cancer), we created an absorbing state specifically for ‘other-cause mortality’ (OCM) and incorporated the probability of OCM when we computed the transition probabilities for each health state. We calculated the probability of OCM specific to PWID by calculating the mortality rate (i.e., number of cases per 1000 population) for each age group (e.g., 20–24, 25–29, etc.) using the administrative data.

### Epidemiologic features of simulated cohort

We created two synthetic cohorts with 100,000 Quebec adults (age range: 18–65 years) who were either ‘Healthy’ or had SSTVI according to the observed prevalence of SSTVI in the province. We used one cohort to simulate a scenario with NSP, and the other cohort to simulate a scenario without NSP. We created our cohorts based on the distributions of demographic characteristics of PWID and injection risk behaviour characteristics from the INSPQ surveillance of the PWID [46] (Table 1).

**Table 1:** List of parameters, distributions, and sources

Parameters	Values	Distribution	Source
Number of cycles^a^	260 weeks		
Cycle length	1 week		
Annual discount rate (costs)	1.5%	Sensitivity analysis (0–3%)	[56]
Annual discount rate (utilities)	1.5%	Sensitivity analysis (0–3%)	[56]
Number of PSA samples	1000		
Weekly incidence proportion of SSTVI (median)	0.001452	5%: 0.00097 95%: 0.00296	Administrative data
Weekly probability of SSTVI (median)	0.001458	5%: 0.00097 95%: 0.00302	Administrative data
PWID Characteristics			
% PWID who used needles/syringes used by someone else	19.6	Beta (1709, 7009)	[46]
% PWID reusing their own needles	29.7	Beta (77, 182)	[72]
% of injections that involved use of needles-syringes that someone else used			[46]
None	85.0	6410 out of 7546	
1–20%	10.1	766 out of 7546	
21–100%	4.9	370 out of 7546	
% Male	62		Administrative data
Years of injection drug use (%)			Administrative data
< 2 years	40.12		
2- < 5 years	35.55		
5- < 8 years	18.16		
8 or more years	6.16		
Injection frequency in the past month (%)			[46]
Never	12.9	1136 out of 8787	
Not every week	22.2	1949 out of 8787	
1–2 days a week	15.6	1366 out of 8787	
3–6 days a week	14.4	1270 out of 8787	
Every day	34.9	3066 out of 8787	
Number of injections in the past month (%)			[46]
1–4	16.9	1268 out of 7519	
5–10	14.2	1067 out of 7519	
11–20	12.3	925 out of 7519	
21–40	10.0	750 out of 7519	
41–60	9.4	704 out of 7519	
61–100	11.1	833 out of 7519	
101–200	15.0	1125 out of 7519	
201–9000	11.3	847 out of 7519	
Risk of SSTVI			
Male (versus Female)	1.14	95% CI = 1.08–1.19	Administrative data
Age			Administrative data
< 25	REF		
25–44	1.19	95% CI = 1.15–1.23	
45+	1.07	95% CI = 1.03–1.11	
Needle sharing	3.31 (IRR)	95% CI = 2.04–5.37	[18]
Reusing needles/syringes	2.1 (OR)	95% CI = 1.2–3.7	[16]
Injection frequency			[16]
Once per week	REF		
2–7 times a week	2.1 (OR)	95% CI = 1.1–4.0	
More than once a day	3.1 (OR)	95% CI = 1.7–5.5	
Number of attempts to achieve injection			[16]
1	REF		
2	2.6 (OR)	95% CI = 2.0–3.5	
3	3.7 (OR)	95% CI = 2.7–5.2	
4+	3.8 (OR)	95% CI = 2.8–5.1	
Years of injection drug use^b^			[19]
< 1 year	REF		
2–4 years	2.49 (OR)	95% CI = 1.16–5.34	
5–7 years	3.95 (OR)	95% CI = 1.73–9.02	
8–10 years	4.84 (OR)	95% CI = 2.14–10.92	
Needle and syringe program			
Risk of sharing injection equipment	0.42 (RR)	95% CI = 0.25–0.72	[34]
Risk of reusing syringes	0.79 (RR)	95% CI = 0.66–0.95	[34]
Costs			
NSP	$322.07	Annual cost of NSP per person in 2022 CAD	[61]
Purulent SSTVI			Administrative data
Outpatient	$100.78	Scale = 76.28 Shape = 1.32	
ED	$1135.70	Scale = 91.54 Shape = 12.41	
Inpatient	$6022.35	Scale = 5994.01 Shape = 1.00	
Inpatient complications	$42,526.40	Scale = 70,215.26 Shape = 0.61	
Non-purulent SSTVI			Administrative data
Outpatient	$93.73	Scale = 72.40 Shape = 1.29	
ED	$1144.64	Scale = 100.93 Shape = 11.34	
Inpatient	$6821.06	Scale = 6119.28 Shape = 1.11	
Inpatient complications	$41,016.23	Scale = 66,790.89 Shape = 0.61	
Utilities			
PWID multiplicative factor	0.9		[73]
Ambulatory Care			
Cellulitis	0.97607		[52, 53]
Abscess	0.97607		[52, 53]
Hospitalization			
Abscess	0.642		[52]
Cellulitis	0.642		[52]

*CAD* Canadian dollar, *IRR* Incidence rate ratio, *NSP* Needle and syringe program, *OR* Odds ratio, *PSA* Probabilistic sensitivity analysis, *PWID* People who inject drugs, *QALY* Quality-adjusted life-year, *REF* Reference, *RR* Risk ratio, *SSTVI* Skin, soft tissue, and vascular infections

^a^The time horizon for this study was 5 years, and we assumed that each year had 52 weeks

^b^Due to the nature of administrative data, there remained uncertainty in capturing the duration of injection drug use among individuals identified as PWID. We assumed that PWID identified in the administrative databases had initiated injection drug use 6 months before being captured in the data. This resulted in small percentage of individuals in the ‘ < 1 year of injection drug use’ category. To account for this, we set ‘ < 2 years’ of injection drug use as the reference category. Based on patient information from the administrative data, we created the remaining categories of duration of injection drug use as ‘2–5 years’, ‘5–8 years’, and ‘> 8 years’ for the analysis

Note: The results in this Table, whose source is ‘administrative data’, were compiled using data from the © Government of Québec (Research file publication date: 2009–2019; Data use approval date: 20 September 2021)

During the data generation step, we based the proportion of PWID who share and reuse needles on the probabilities of needle sharing and reusing captured in INSPQ surveillance data [46]. We used the risk ratio estimates corresponding to the effectiveness of NSP on sharing and reusing needles [34] and multiplied the inverse of these risk ratios to derive the probabilities of sharing and reusing needles under the scenario without NSP. Consequently, the proportion of PWID who shared and reused needles was higher without NSP, leading to a higher probability of acquiring SSTVI in the cohort without NSP compared to the cohort with NSP.

To estimate the prevalence of SSTVI and transition probabilities, we used Quebec’s provincial administrative health databases (January 2009-December 2019) with a validated algorithm to identify PWID (sensitivity: 0.85, specificity: 0.80) [54]. We then searched the records of physician visits, ED visits, and hospital admissions in the administrative health databases between January 1, 2009 and March 31, 2019 to identify cases of SSTVI (see Supplementary Materials).

To derive the individual probability of SSTVI, we estimated the proportion of the general population (including individuals who do not use drugs) with SSTVI between January 2009 and March 2019 using the above administrative health databases. We set this value as the “base” probability of SSTVI if the PWID did not inject in the past month, did not share needles, and did not reuse injection equipment. We then included relative risks of SSTVI occurrence associated with injection risk behaviour (e.g., injection frequency, sharing injection equipment, or reusing needles and syringes) [16, 18, 19] and changes in injection risk behaviour associated with the implementation of NSP [34]. For PWID with behavioural risk factors, this “base” probability was multiplied by the “risk multiplier” (i.e., risk ratios or odds ratios from the literature; see Supplementary Materials for more detail).

### Cost parameters

We assessed the direct healthcare costs from the public payer perspective (Quebec Ministry of Health and Social Services), which were captured through the provincial public system. We report all costs in 2022 Canadian dollars after adjusting for inflation using Statistics Canada’s Consumer Price Index for Quebec (Health and Personal Care Products) [55]. We used records of physician visits, ED visits, and hospital admissions to derive cost estimates at each stage of SSTVI treatment. Complications during episodes of hospitalization were based on the length of hospital stay and record of procedure codes. We assumed that hospitalization longer than 7 days with a record of surgical procedures constituted complication in inpatient settings, which we designated as the ‘inpatient complication’ health state. To calculate the cost of SSTVI in each treatment setting (e.g., outpatient, ED, and inpatient), we took the average cost of treating the case at each of the settings for each type of SSTVI, which we derived from the administrative data (details described in Supplementary Materials).

Due to the lack of data on the cost of NSP in Quebec, we adjusted the annual cost of NSP from Ontario to account for the difference in population between these two provinces. Since NSP are available to all PWID, we divided the total cost of NSP over the 5-year period by the number of individuals in the simulation and the number of weeks during the same period to derive the weekly average cost of NSP per PWID.

We applied the discount rate of 1.5% for costs and health outcomes (with 0% and 3% as sensitivity analyses) in accordance with the Canadian Agency for Drugs and Technologies in Health’s recommendations [56]. More specific assumptions and algorithms used to create the input parameters are enumerated in the accompanying Supplementary Materials.

### Analysis

We conducted a cost-utility analysis of a needle and syringe program, in which we computed the incremental cost-effectiveness ratio (ICER) to derive the cost per quality adjusted life year (QALY). In addition, we calculated the specific healthcare costs for each treatment setting (i.e., location of care) and type of SSTVI to estimate the economic burden in each scenario.

Relatedly, we conducted a recurrent-event survival analysis on data from the microsimulation output (details in Supplementary Materials), in which we estimated the risk of recurrent outpatient visits, emergency department visits, and hospitalizations associated with NSP compared to not having an NSP. We used the marginal means model [57], an extension of the Cox proportional hazards model, to estimate the hazard ratios (HR) and 95% confidence intervals (CI) of the association between NSP and the risk of recurrent use of the healthcare system for treating SSTVI. We chose to run the marginal means model because multiple episodes of the outcome over the course of follow-up from the same individual could be correlated. In addition, the marginal approach provides a population-level estimate of the cumulative hazard, which could then be used to calculate the expected number of events that the individual experienced up to a given time [58]. Where the within-subject correlation is complex and unknown, the marginal means model can account for it in a flexible and unbiased manner (i.e., the robust standard error is valid even when the correlation dependence structure is mis-specified) [59].

To estimate the effect of NSP on the likelihood of SSTVI mortality, we ran competing risk survival analysis. We chose this analytic framework to account for the presence of competing events (e.g., other-cause mortality). We constructed the event-specific cumulative incidence function and ran the Fine-Gray model for competing risk regression [60], as it enables interpretation of hazard ratios similar to a Cox proportional hazards model.

Finally, we conducted a probabilistic sensitivity analysis (PSA) with 1,000 model runs on 10,000 individuals (for each treatment strategy). For parameters with uncertainty (e.g., costs, QALY, and risk ratios), we assigned a statistical distribution (e.g., beta, gamma, or lognormal) in which its mean value was considered the base case scenario. We then computed the quantile-based credible intervals at the 2.5th and 97.5th percentiles to reflect the range of values from the PSA iterations. Based on the PSA outputs, we constructed a PSA scatter plot and fitted the cost-effectiveness acceptability curves to determine the probability of cost-effectiveness of NSP. We derived the model parameters (e.g., transition probabilities and costs) from the administrative data using SAS 9.4. We then conducted model simulation and statistical analyses in R version 4.2.3.

## Results

**Fig. 2:**
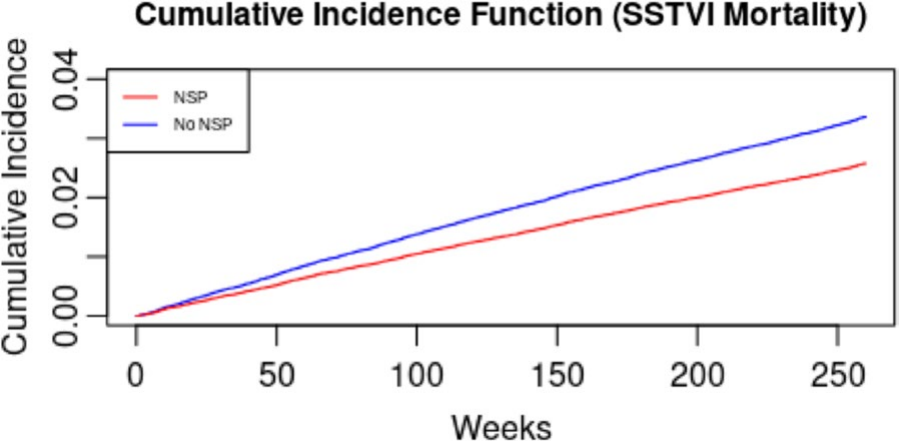
Cumulative incidence function for SSTVI mortality with and without NSP. Note: The results in this figure were compiled using data from the © Government of Québec (Research file publication date: 2009–2019; Data use approval date: 20 September 2021)

Tables 2 and 3 display the results from analyses of the public health burden of SSTVI. We estimated that NSP had a protective effect against SSTVI mortality, with 788 fewer deaths per 100,000 PWID (No NSP: 3,360 deaths from SSTVI vs. With NSP: 2,572 deaths from SSTVI in simulated cohort of 100,000). This corresponded to hazard ratio of 0.76 (95% CI = 0.72–0.80; Fig. 2). Upon examination of health service use, we estimated that health service utilization over the 5-year period remained lower under the scenario with NSP (outpatient: 66,511 vs. 86,879; ED: 9920 vs. 12,922; inpatient: 4282 vs. 5596). Relatedly, NSP was associated with modest reduction in the relative hazard of recurrent outpatient visits (HR = 0.96; 95% CI = 0.95–0.97) for purulent SSTVI as well as outpatient (HR = 0.88; 95% CI = 0.87–0.88) and ED visits (HR = 0.98; 95% CI = 0.97–0.99) for non-purulent SSTVI. However, the hazard of recurrent inpatient SSTVI (including complications) remained similar across the two cohorts.

**Table 2:** SSTVI mortality and health service use with and without NSP (95% CI)

Parameters	Number of Events^a^		Incidence Rates^b^	
	With NSP	No NSP	With NSP	No NSP
SSTVI Mortality	2572 (2475–2672)	3360 (3250–3474)	5.57 (5.36–5.79)	7.31 (7.06–7.56)
OP Visits	66,511 (66,217–66,803)	86,879 (86,668–87,087)	147.00 (145.97–148.04)	192.84 (191.69–194.00)
ED Visits	9920 (9736–10,107)	12,922 (12,715–13,132)	21.93 (21.50–22.36)	28.68 (28.20–29.17)
IP Visits	4282 (4158–4410)	5596 (5455–5741)	9.46 (9.19–9.75)	12.42 (12.10–12.75)

*CI* confidence interval, *ED* emergency department, *IP* inpatient, *NSP* needle and syringe programs, *OP* outpatient, *PWID* people who inject drugs, *SSTVI* skin, soft tissue, and vascular infections

^a^The number of events refers to the total number of deaths or contacts with the healthcare system per 100,000 PWID over a 5-year period

^b^Incidence rates were calculated as the number of events per 1000 population for SSTVI mortality and the number of events per 1000 person-years for contacts with the healthcare system

Note: The results in this table were compiled using data from the © Government of Québec (Research file publication date: 2009–2019; Data use approval date: 20 September 2021)

**Table 3:** Hazard ratios for SSTVI mortality and health service use with NSP

Mortality	HR^a^ (95% CI)	Purulent	HR^b^ (95% CI)	Non-Purulent	HR^b^ (95% CI)
SSTVI Mortality	0.76 (0.72–0.80)	OP	0.96 (0.95–0.97)	OP	0.88 (0.87–0.88)
		ED	0.99 (0.98–1.01)	ED	0.98 (0.97–0.99)
		IP	0.99 (0.97–1.01)	IP	0.99 (0.98–1.00)
		IPC	1.00 (0.98–1.02)	IPC	1.00 (0.98–1.01)
		PDD	1.00 (0.96–1.05)	PDD	0.99 (0.96–1.02)

*CI* confidence interval, *ED* emergency department; HR = hazard ratio; IP = inpatient; IPC = inpatient complications; NSP = needle and syringe programs; OP = outpatient; PDD = patient-directed discharge; SSTVI = skin, soft tissue, and vascular infections

^a^For SSTVI mortality, we conducted a competing risk analysis using the Fine-Gray model to derive the hazard ratio

^b^For health service use, we conducted a recurrent event analysis using the marginal mean model to derive the hazard ratio

Note: The results in this table were compiled using data from the © Government of Québec (Research file publication date: 2009–2019; Data use approval date: 20 September 2021)

Table 4 summarizes the results of the cost-utility analysis, with discounted cost and QALY estimates. When 1.5% discount rate was assumed, the incremental cost under the NSP scenario compared to the no NSP scenario was $1,207 with 0.017 QALY gains, which correspond to an ICER of $70,278 per QALY. At 0% and 3% discount rates, we also observed similar increase in costs and QALYs with an ICER of $69,455 per QALY and $71,108 per QALY, respectively. When we assumed different scenarios for the annual cost of NSP per PWID ($300–$400 per person), the ICER ranged from $64,103 per QALY to $92,333 per QALY.

**Table 4:** Estimates of cost, QALY, and ICER with and without NSP

Scenario	Intervention	Cost	MCSE	QALY	MCSE	INC Cost	MCSE	INC QALY	MCSE	ICER
Base Case	No NSP	$919	14	3.939	0.003					
	NSP	$2125	12	3.956	0.003	$1207	7	0.017	0.001	$70,278/QALY
Discount Rate 0%	No NSP	$945	14	4.052	0.003					
	NSP	$2187	13	4.07	0.003	$1241	7	0.018	0.001	$69,455/QALY
Discount Rate 3%	No NSP	$894	14	3.832	0.003					
	NSP	$2068	12	3.849	0.003	$1174	7	0.017	0.001	$71,108/QALY
Annual NSP Cost^a^ ($300)	No NSP	$919	14	3.939	0.003					
	NSP	$2019	12	3.956	0.003	$1100	7	0.017	0.001	$64,103/QALY
Annual NSP Cost^a^ ($350)	No NSP	$919	14	3.939	0.003					
	NSP	$2262	12	3.956	0.003	$1343	7	0.017	0.001	$78,218/QALY
Annual NSP Cost^a^ ($375)	No NSP	$919	14	3.939	0.003					
	NSP	$2383	12	3.956	0.003	$1464	7	0.017	0.001	$85,276/QALY
Annual NSP Cost^a^ ($400)	No NSP	$919	14	3.939	0.003					
	NSP	$2504	12	3.956	0.003	$1585	7	0.017	0.001	$92,333/QALY

*ICER* incremental cost-effectiveness ratio, *INC* incremental, *MCSE* Monte Carlo standard error, *NSP* needle and syringe programs, *QALY* quality-adjusted life-years

^a^NSP costs refer to the annual cost per person who injects drugs

Note: The results in this table were compiled using data from the © Government of Québec (Research file publication date: 2009–2019; Data use approval date: 20 September 2021)

**Fig. 3:**
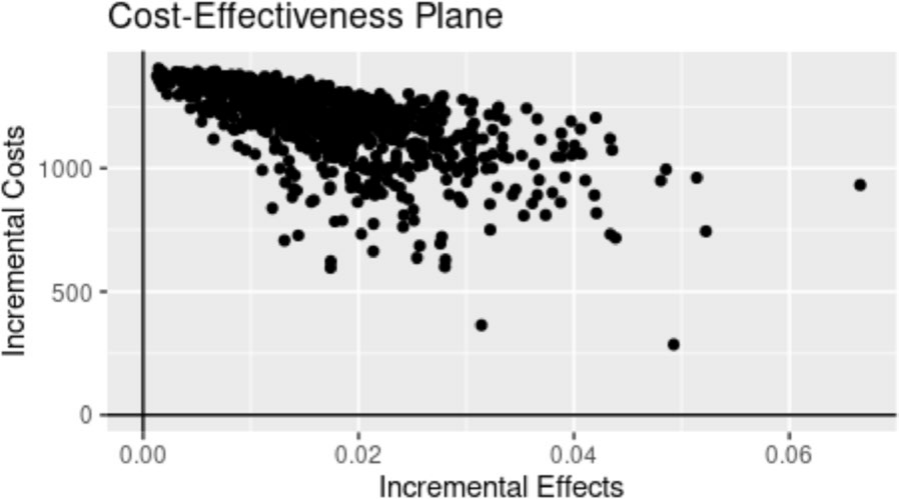
Cost-effectiveness plane of incremental costs and incremental QALY from probabilistic sensitivity analysis with 1,000 iterations of the simulation. Note: The results in this figure were compiled using data from the © Government of Québec (Research file publication date: 2009–2019; Data use approval date: 20 September 2021)

**Fig. 4:**
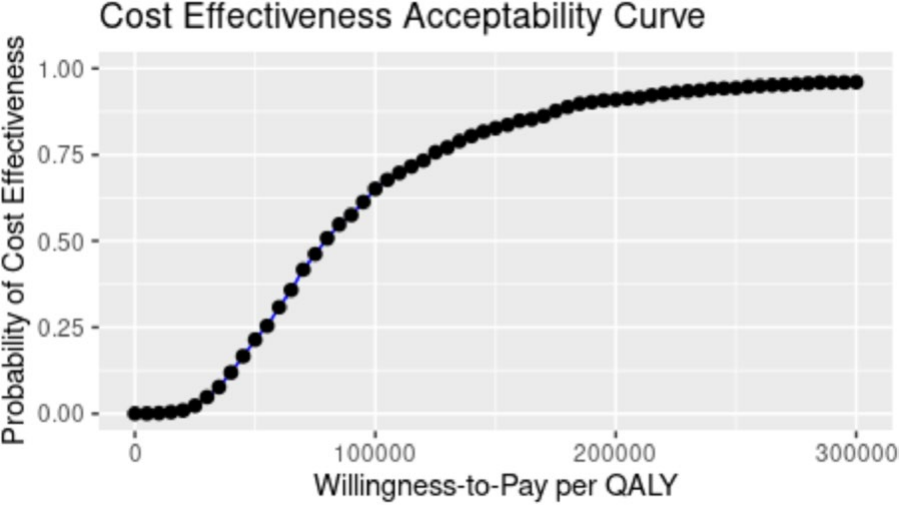
Cost-effectiveness acceptability curve with the probability of cost-effectiveness of NSP by willingness-to-pay thresholds from probabilistic sensitivity analysis with 1,000 iterations of the simulation. Note: The results in this figure were compiled using data from the © Government of Québec (Research file publication date: 2009–2019; Data use approval date: 20 September 2021)

Figures 3 and 4 characterize the results of the PSA. Figure 3 illustrates the cost-effectiveness plane, in which we plotted the incremental cost and incremental effectiveness for each of the 1,000 PSA runs. All 1,000 points lie in the quadrant corresponding to higher effectiveness and higher costs. Figure 4 depicts the cost-effectiveness acceptability curve corresponding to different willingness-to-pay thresholds ranging from $0 to $300,000 per QALY. If the decision maker is willing to pay $100,000 per QALY, the probability of cost-effectiveness associated with NSP is 65.1%. Similarly, if the decision maker is willing to pay $200,000 per QALY, then there is 90.9% probability that NSP remains cost-effective.

## Discussion

To our knowledge, this is the first study to evaluate cost-effectiveness of needle and syringe programs in relation to skin, soft tissue, and vascular infections for people who inject drugs. In our model, we assessed the epidemiologic impact of NSP on the burden of SSTVI in PWID as well as economic implications of SSTVI on the Quebec healthcare system compared to a counterfactual scenario had NSP not been implemented. We estimated that having NSP led to fewer SSTVI deaths over the five-year period compared to not having NSP, with lower relative hazard of SSTVI mortality. Relatedly, under the scenario with NSP, there were fewer contacts with the healthcare system as well as lower hazards of recurrent outpatient visits and ED visits to treat SSTVI.

Our results provide evidence that complements earlier Canadian studies on harm reduction strategies that demonstrated their cost-effectiveness [39–42, 61]. An earlier economic modelling study of NSP in Ontario concluded that NSP was associated with 359 new HIV infections averted ($106,928 per disability-adjusted life-year averted) [61]. Other studies have focused on the cost-effectiveness of supervised consumption sites, which also distribute needles and injection equipment. These latter studies have concluded cost-effectiveness based on prevention of HIV and HCV, which resulted from reduction in high-risk injection behaviours and increased uptake of safer injection practices [41, 42, 62]. Our findings remain significant because we observed that NSP reduce the epidemiological and economic burdens of SSTVI. In addition to existing evidence of effectiveness of NSP against blood-borne infections, our study highlights complementing benefits of NSP by demonstrating their clinical and cost-effectiveness in addressing bacterial infections.

The relatively high ICER observed should be interpreted with caution because the higher ICER could in large part be due to the lower rate of mortality from SSTVI under the ‘With NSP’ scenario compared to the ‘No NSP’ scenario. Under the former scenario, there were more individuals who were alive. This meant that there were a greater number of individuals who were at risk of health service use as well as those who incurred the NSP costs (i.e., fixed costs needed to operate the programs) at each cycle under the ‘With NSP’ scenario. In other words, by virtue of saving lives, having the NSP resulted in higher direct healthcare costs among those alive, which in turn increased the incremental costs for the healthcare system. In addition, the incremental QALY gains from having NSP remained very small, which then resulted in higher incremental cost per incremental QALY (i.e., ICER).

Despite the relatively high ICER, other important benefits of the NSP include lower recurrent health service use under the ‘With NSP’ scenario. The observed differences in health service utilization patterns could be due to how NSP operate in Quebec. Some of the NSP in the province (also known as ‘injection equipment access centres’) provide access to medical care and treatment (e.g., wound care to treat injection-related wounds) [63]. In addition, NSP in Quebec are often operated by community organizations unaffiliated with the healthcare system [64], which helps establish trust with the PWID population [65]. With high level of trust between clients and NSP staff [66], PWID may utilize NSP as an alternative to physician visits when seeking medical care for SSTVI, and NSP may bridge the gap between medical care and PWID.

In addition to the health benefits to individuals, the lower hazard of recurrent SSTVI associated with NSP has important implications on the Quebec health care system. The lower hazard ratios associated with NSP suggest that they may not only operate as a lower cost alternative to formal medical service but also as a preventive strategy against SSTVI for PWID. However, our results are to be interpreted with caution. The findings of recurrent-event analysis were from a simulated cohort (i.e., synthetic data). The model relied on simplified assumptions of the cohort of PWID, and the model assumptions may not have accurately reflected the complexities of the real-world patterns of SSTVI incidence and recurrence. While this could serve as preliminary evidence on the benefits of NSP against recurrent SSTVI, evidence from real-world data may be needed to strengthen the current findings and to inform decision making.

Other benefits to the healthcare system may include reduction in early departures from healthcare settings (i.e., patient-directed discharge). Owing to a small number of early departures from medical institutions observed in our simulation (i.e., rare outcome), we were only able to see a modest reduction in the number of patient-directed discharges. However, NSP mitigate needle sharing and reuse of injection equipment [34–38], which are factors that elevate the risk of early departures among PWID [67, 68]. Further, community-level treatment and care could reduce the risk of self-directed discharges from hospitals and emergency departments [69]. Many NSP in Quebec operate at the community level outside the healthcare system [64], and NSP coverage in the province is high at 82% [70]. With expanded operation, NSP may contribute to additional reduction in hospital re-admissions that arise from complications and worsened health outcomes following patient-initiated early departures [71].

### Strengths

First, our model framework captured the real-world complexities surrounding individuals’ unique clinical pathways for the treatment of SSTVI. Second, we used Quebec administrative health data and extensive public health surveillance on PWID conducted by the INSPQ to inform our model parameters. The administrative databases allowed our model inputs to reflect the health service utilization patterns of PWID and current costs and burdens of SSTVI in Quebec’s healthcare system in each care setting (e.g., outpatient, ED, and inpatient). Relatedly, the INSPQ surveillance data captured the most up-to-date population characteristics of PWID, which are not readily available in traditional administrative databases. Third, in addition to the economic evaluation, we conducted survival analysis that accounts for recurrent events and competing risks, enabling the assessment of both cost-effectiveness and clinical effectiveness of NSP. Thus, our study provides a more comprehensive overview of the comparative effectiveness between the ‘NSP’ and the ‘No NSP’ scenarios.

### Limitations

First, our model may not have adequately captured the variabilities in real-world clinical practice, as we assumed that the quality of care delivered, and health system performance remained the same over the course of follow-up for both cohorts. To mitigate this concern, we varied the risk ratios corresponding to injection risk behaviours as well as the cost and utility parameters in our PSA, where the intervention and the control cohorts had different values for these parameters at each iteration of the PSA. Second, due to the lack of availability of NSP costs in Quebec, we used the NSP cost estimates from Ontario. In addition, we were unable to divide NSP costs into fixed cost and variable cost components and how NSP affected each component due to the lack of available information beyond the total cost of operating the NSP. While we were unable to model the effect of NSP on specific cost components, we conducted deterministic sensitivity analyses to mitigate concerns surrounding the NSP costs per PWID in Quebec by re-running the model with five different estimates of NSP cost per PWID per year. Third, due to a lack of Quebec-specific information on the effectiveness of NSP against injection risk behaviours, we relied on the risk ratio estimates derived from epidemiological studies conducted in other parts of the world. This may raise concerns around the validity of model findings due to discrepancies between the sources of the estimates and the Quebec regional contexts. To mitigate these concerns, we calibrated our model probabilistically, which accounted for parameter uncertainties by generating posterior distributions that reflect the range of plausible values for the risk ratio estimates. Fourth, there was uncertainty around the extent to which multiple injection risk behaviours interact and elevate the risk of SSTVI. To account for this uncertainty, we conducted Bayesian model calibration using the sample importance resampling approach, which generated a posterior set of risk ratio estimates based on 659 unique sets of risk ratios (details of model calibration in Supplementary Materials).

## Conclusion

Needle and syringe programs are effective in preventing and reducing transmission of SSTVI. Compared to not having NSP, having NSP is associated with reduced hazard of SSTVI mortality as well as lower relative hazard of recurrent contacts with the healthcare system for treating SSTVI. Expansion of NSP services may maximize their benefits and further reduce harms associated with SSTVI in PWID.

## Supplementary Information

Supplementary Material 1.

## Data Availability

The administrative data that support the findings of this study are available only through the CADRISQ site, secure research environment accessible only to accredited researchers in Quebec for research purposes. Therefore, restrictions apply to the availability of these data, and data requests must be made with permission from the *Institut de la Statistique du Québec*. The R code used in the current study is available in the GitHub repository (URL: https://github.com/jihoon-jay/Microsimulation).

## Abbreviations

CAD: Canadian dollar
CADRISQ: Centre d’accès aux données de recherche de l’Institut de la statistique du Québec
CCI: Canadian Classification of Health Interventions
CI: Confidence interval
CMG: Case mix group
DAA: Discharge against medical advice
DIN: Drug Identification Number
ED: Emergency department
HCV: Hepatitis C virus
HR: Hazard ratio
I&D: Incision and drainage
ICD: International Classification of Diseases
ICER: Incremental cost-effectiveness ratio
ICU: Intensive care unit
IDSA: Infectious Disease Society of America
INC: Incremental
INSPQ: Institut national de la santé publique du Québec/Quebec Provincial Public Health Institute
IP: Inpatient
IPC: Inpatient complications
IRR: Incidence rate ratio
ISQ: Institut de la statistique du Québec
MCSE: Monte Carlo standard error
NSP: Needle and syringe programs
OCM: Other cause mortality
OP: Outpatient
OR: Odds ratio
PSA: Probabilistic sensitivity analysis
PWID: People who inject drugs
QALY: Quality adjusted life years
REF: Reference
SSTVI: Skin, soft tissue, and vascular infections
TMP-SMX: Trimethoprim-sulfamethoxazole
UCSF: University of California San Francisco

## Footnotes

1 Une partie de la compilation est effectuée à partir de données provenant du © Gouvernement du Québec (année de la publication du Fichier de recherche: 2009–2019).

2 Le Gouvernement du Québec n’est pas responsable des compilations ni de l’interprétation des résultats produits à l’aide du Fichier de recherche.
