# Correction: Economic evaluation of the effect of needle and syringe programs on skin, soft tissue, and vascular infections in people who inject drugs: a microsimulation modelling approach

**DOI:** 10.1186/s12954-024-01083-x

**Published:** 2024-09-09

**Authors:** Jihoon Lim, W Alton Russell, Mariam El-Sheikh, David L. Buckeridge, Dimitra Panagiotoglou

**Affiliations:** Department of Epidemiology, Biostatistics, and Occupational Health, McGill University, 2001 McGill College Avenue, Suite 1200, Montreal, QC H3A 1G1 Canada

**Correction: Harm Reduction Journal (2024) 21:126**

10.1186/s12954-024-01037-3

Following publication of the original article [1], the author would like to include missing co-author W Alton Russell in the author group. The author contribution is mentioned in the acknowledgement section, however inadvertently not provided in the author group.

The author group should appear as mentioned below:

Jihoon Lim^1^, W Alton Russell^1^, Mariam El Sheikh^1^, David L Buckeridge^1^ and Dimitra Panagiotoglou^1*^

The original article has been corrected.
