# Supplementary material for: Economic evaluation of the effect of needle and syringe programs on skin, soft tissue, and vascular infections in people who inject drugs: a microsimulation modelling approach
Source: Harm Reduct J. 2024 Jun 28;21:126. doi: 10.1186/s12954-024-01037-3 (PMC11212409; doi:10.1186/s12954-024-01037-3)
Supplement: Supplementary file 1 — Supplementary Material 1. [file 12954_2024_1037_MOESM1_ESM.docx]

**Economic evaluation of needle and syringe programs on skin, soft tissue, and vascular infections in people who inject drugs: A microsimulation modelling approach**

Jihoon Lim, MS; Mariam El-Sheikh, MSc; David L. Buckeridge, MD PhD; Dimitra Panagiotoglou, PhD

Note: All Tables and Figures in the Supplementary Materials below were compiled using data from the © Government of Québec (Research file publication date: 2009-2019; Data use approval date: 20 September 2021). All inferences, opinions, and conclusions drawn in this publication are those of the authors, and the © Government of Québec is not responsible for the compilations or the interpretation of the results produced using the research files.

Note: Une partie de la compilation est effectuée à partir de données provenant du © Gouvernement du Québec (année de la publication du Fichier de recherche: 2009-2019). Le © Gouvernement du Québec n’est pas responsable des compilations ni de l’interprétation des résultats produits à l’aide du Fichier de recherche.

**Appendix A: Model Framework**

*Data Sources*

The six administrative databases from Quebec included : (1) *Maintenance et exploitation des données pour l’étude de la clientèle hospitalière* (“Hospitalizations”), (2) *Services médicaux rémunérés à l’acte* (“Physician Claims”), (3) *Banque de données communes des urgences* (“ED visits”), (4) *Services pharmaceutiques* (“Public prescription drug plan”), (5) *Bureau du Coroner du Québec* (“Vital Statistics/Mortality”), and (6) *Registre des événements démographiques* (“Registered persons file”).

Datasets 1-4 include records of hospitalizations, physician visits, ED visits, and drug dispensation, respectively. These data were used to identify PWID, ascertain SSTVI, calculate transition probabilities, and derive costs associated with the healthcare encounter and procedure. Dataset 5 includes records of mortality, and it was used to capture deaths from SSTVI complications, such as infective endocarditis or surgical site infections. Dataset 6 contains age, sex, and socioeconomic status variables, which were used to describe the distribution of demographic characteristics of the study population.

*Health States*

The microsimulation model had 13 health states. These include: Healthy; SSTVI; self-treatment death (i.e., mortality from SSTVI but without prior history of contact with the healthcare system); purulent SSTVI (outpatient, ED, inpatient, inpatient complications); non-purulent SSTVI (outpatient, ED, inpatient, inpatient complications); death from SSTVI; and other-cause mortality.

*Study Population*

For all calculations of probabilities and costs associated with skin, soft tissue, and vascular infections (SSTVI) using Quebec provincial administrative health databases, we restricted the study population to people who inject drugs (PWID) between the ages of 18 and 65 years.

*Definition of Skin, Soft Tissue, and Vascular Infections*

Purulent SSTVI included cases of abscess, furuncle, carbuncle, and folliculitis (ICD-9 codes: 680*, 681*, 682*, and 704.8* and ICD-10 code: L02* and L66.2*). Non-purulent SSTVI included cases of cellulitis, erysipelas, and necrotizing fasciitis (ICD-9 codes: 681*, 682*, and 72886 and ICD-10 codes: L03*, M726*, and A46*). For outpatient cases only, physicians in Quebec are not legally required to input ICD-9 diagnostic codes when submitting claims. Where the ICD-9 codes were missing, we used the grouping codes and act codes to capture SSTVI cases. The grouping codes included: 196 (flat fee/pricing for microbiology and infectiology), 760 (diagnostic acts in the laboratory service within an institution’s microbiology unit), 838 (excision or surgical incision of abscess), 842 (ablation of hyperplastic tissues and excess mucus), and 852 (reparation of soft tissue laceration). We searched all the 5-digit act codes in the physician claims that corresponded to incision and excision to identify procedures done specifically to treat abscess and perform drainage. To distinguish between purulent (abscess) and non-purulent (cellulitis) SSTVI in physician claims (ICD-9), we looked at records of antibiotics prescribed from the date on which the patient record showed up in physician claims. Based on the Infectious Diseases Society of America (IDSA) and University of California San Francisco (UCSF) guidelines [1, 2], we categorized SSTVI into purulent cases if they involved incision and drainage (I&D) and non-purulent cases otherwise. I&D procedures conducted in outpatient settings were identified using the procedure category ‘838’ (excision or surgical incision for abscess) or the following act codes:

- Incision: 02044, 02045, 02026, 02775, 02776, 02682, 02697, 02698, 02699, 02704, 02269, 02564, 02574, 02875, 02876, 02877, 02878, 09517, 09561, 09562, 03000, 03001, 03003, 03020, 03032, 03033, 03034, 03023, 03038, 03026, 03028, 03120, 03123, 03029, 03017, 03030, 03031, 05001, 05002, 05004, 05100, 05101, 05006, 05007, 05008, 05009, 05021, 05085, 05086, 05087, 05012, 05013, 05040, 05041, 05042, 05044, 05045, 05010, 05011, 05019, 05077, 05118, 05191, 05191, 05195, 05196, 05080, 05081, 05073, 05083, 05084, 05096, 05482, 05483, 05484, 05487, 06022, 06030, 06031, 06032, 06033, 06034, 06394, 06064, 06043, 06062, 06076, 07538, 07557, 07558, 07559, 07560, 07561, 07562, 07563, 07564, 07565, 07566, 07567, 07568, 07682, 07698, 07699, 07683, 07713, 07714, 07063, 07065, 07066, 07067, 07071, 07072, 07076, 00124, 00324, 09472, 00508, 09473, 09474, 01013, 01014, 01015, 01016, 01017, 01024, 01018, 01019, 01020, 18088, 18108, 18112, 09517, 09561, 09562, 02008, 02012, 02014, 02015, 09597, 02327, 05016, 05017, 05018, 05003, 05036, 05050, 05052, 05057, 05059, 07065, 07068, 07071, 07072, 07075, 07076, 00418, 20224, 20225, 00124, 00324, 00298, 00299, 00178, 20598, 09472, 20075, 09474, 00491, 01011, 01005, 01006, 01001, 01002, 01003, 01004, 01007, 01008, 01023, 01009
- Excision: 03101, 03102, 03160, 03172, 03173, 03161, 03199, 03200, 03201, 03207, 03208, 03209, 03239, 03122, 03140, 03124, 03125, 03126, 03127, 03128, 03129, 03130, 03131, 03132, 03133, 03134, 03135, 03136, 03137, 03138, 03141, 03139, 03232, 03252, 01201, 01250, 01205, 01251, 01252, 01228, 01230, 01037, 01038, 01231, 01232, 01233, 01234, 02212, 02236, 02235, 02256, 02255, 02233, 09534, 09536, 02208, 02209, 02155, 02023, 18170, 02127, 02152, 02153, 02154, 02866, 02867, 02868, 02869, 02948, 02870, 02871, 02596, 02597, 02244, 02241, 02234, 02246, 02242, 02245, 18138, 09593, 02201, 02202, 09537, 02007, 02082, 02083, 03165, 03166, 03167, 03171, 03164, 03169, 03105, 03104, 03215, 03368, 03369, 03110, 03111, 03112, 03113, 03108, 03210, 03211, 03246, 03235, 03236, 03237, 03238, 03247, 03248, 03249, 03250, 03109, 05173, 05161, 05034, 05035, 05158, 05159, 05168, 05208, 05203, 05014, 05066, 05068, 05065, 05069, 05070, 05514, 05120, 05124, 05177, 05288, 05015, 05126, 05212, 05172, 05502, 05503, 05178, 05198, 05170, 05213, 05171, 05043, 05201, 05209, 05192, 05182, 05536, 05183, 05537, 05246, 05250, 05240, 05538, 05241, 05242, 05243, 05244, 05292, 05293, 05294, 05253, 05539, 05540, 05812, 05304, 05144, 05186, 05248, 05239, 05145, 05146, 05295, 05174, 05147, 05148, 05122, 05137, 06190, 06210, 06211, 06035, 06215, 06219, 06106, 06107, 06064, 06170, 06169, 06172, 06189, 06466, 06156, 06157, 06158, 06258, 06356, 06150, 06151, 06152, 06133, 06153, 06180, 06280, 06134, 06281, 06282, 06284, 07713, 07714, 07716, 07717, 07063, 07173, 07167, 07168, 07249, 07174, 07157, 07420, 07421, 07480, 07467, 07248, 07250, 07813, 07150, 07185, 07184, 07197, 07198, 07255

*Treatment Settings*

We defined an episode of SSTVI as repeated claims that occur within 7 days of each other [3]. In other words, these repeated claims constituted the same episode of SSTVI. We defined an outpatient case as a record physician claim with no additional claims for ≥ 7 days. Similarly, we defined an emergency department (ED) case as a record of ED visit with no additional claims for ≥ 7 days. An inpatient case was defined as a record of hospitalization that followed an outpatient claim or ED claim from ≤ 7 days prior to hospital admission. An inpatient complication refers to an inpatient case, which involved surgical procedures. We identified surgical procedures using the following Canadian Classification of Health Intervention (CCI) procedure codes: Section and Groups 1YA-1YZ, 2YA-2YZ, and 3YL-3YZ (Interventions on the Skin, Subcutaneous Tissue and Breast) with ‘LA’, ‘DA’, ‘CA’, ‘JA’, and ‘HA’ as qualifiers [4, 5].

For individuals who utilized the healthcare system to treat SSTVI, we assumed that hospital admissions occurred only after initial contact with the healthcare system through physician or ED visits. From these two health states, patients could transition to the ‘inpatient’ or ‘inpatient complication’ states due to varying severity of SSTVI. However, we assumed that progression of SSTVI occurred within the same type of SSTVI. In other words, patients who sought outpatient care for purulent SSTVI could be admitted to the hospital for the same purulent case of SSTVI, but they could not be admitted to the hospital for non-purulent SSTVI. Similarly, we assumed that a non-purulent SSTVI could not progress into a purulent SSTVI.

For inpatient cases both with and without surgical procedures, we assumed that hospital re-admission could take place in the next cycle. In such cases, we assumed that patients transitioned to the ‘inpatient’ health state, even though they were in the ‘inpatient complication’ state in the previous cycle.

*Self-Treatment and Mortality*

In the literature, the proportion of SSTVI self-treatment varied widely across studies (range: 32%-86%) [6-9], and none were from the Canadian or Quebec context. In our model, we assumed that 75% of the PWID would self-treat SSTVI. Next, we accounted for the likelihood of self-resolution, delays in seeking care from a doctor, not seeking treatment, and applying self-treatment. The likelihood of self-resolution of SSTVI varied widely (range: 29%-91%) [6, 7], and one study observed that PWID waited on average 5 days before seeking treatment for SSTVI [8]. Taking these factors into account, we assumed that among those who self-treat their SSTVI, the probability of unresolved SSTVI (i.e., P [SSTVI -> SSTVI]) was 65%.

To compute the probability of death from SSTVI (self-treatment), we first searched the inpatient, ED, and outpatient records for each PWID included in the study to identify their last contact with the healthcare system. For individuals who died over the course of their follow-up, we searched the mortality database to look for records whose first five leading causes of death included the following diagnostic codes:

- SSTVI (ICD-10: I80, L97, L988, M793, A480, G06, G09, K630, K650, K750, L02, L03, M5402, M726, N10)
- Infective endocarditis (ICD-10: I33, I38, I39)
- Surgical site infections (ICD-10: T814, T846, T847, and T857)
- Sepsis (ICD-10: A039, A021, A207, A217, A227, A239, A241, A267, A280, A282, A327, A392, A393, A394, A40, A400, A401, A402, A403, A408, A409, A41, A410, A411, A412, A413, A415, A4150*, A4151*, A4152*, A4158*, A418, A4180*, A4188*, A419, A427, B007, B377, P360, P361, P362, P363, P364, P365, P368, P369, P352, P372, P375, A047, B9548, B956, B962, J189, J440, and N390)
- Bacteremia (ICD-10: R7881)
- Osteomyelitis (ICD-10: M86)
- Gangrene (ICD-10: I96)
- Lymphadenitis (ICD-10: L04)
- Myositis (ICD-10: M60)

We assumed that if the individual had no record of health service utilization for at least 15 days before death from SSTVI, then he or she sought self-treatment. We computed the probability of “self-treatment death” as the number of deaths that occurred without contact with the healthcare system for 15 days divided by the number of PWID who were at risk of developing SSTVI.

*SSTVI Mortality*

We computed the probability of SSTVI mortality (following health service utilization) using the same diagnostic coding algorithm as SSTVI mortality (self-treatment). We assumed that SSTVI mortality could take place following ED visits or hospitalizations (both inpatient and inpatient complication cases). Based on these premises, we calculated the probability of transitioning from health service use to SSTVI death by taking the number of SSTVI deaths within 7 days of the ED visit or hospitalization by the number of ED visits or hospitalizations for SSTVI.

*Other-Cause Mortality*

The probability of other cause mortality (OCM) was added to all health states (except self-treatment death and SSTVI death) for each individual. We defined OCM as mortality whose first five leading causes of death were not SSTVI, the complement of the SSTVI mortality that we defined above. We calculated the probability of OCM specific to PWID by calculating the mortality rate (i.e., number of cases per 1,000 population) for each age group (e.g., 20-24, 25-29, etc.) using the administrative data.

*Hospital Re-admissions*

Although there were inpatient and inpatient complication health states for both purulent and non-purulent SSTVI, we assumed that hospital re-admissions took place only via regular inpatient settings. That is, only the IP -> IP transitions were possible. We excluded IPC -> IP transition because upon analysis using administrative data, there were fewer than 5 individuals who underwent this transition. As per regulations dictated by the *Institut de la Statistique du Québec*, we suppressed all frequencies fewer than 5 to mitigate the risk of identification of specific patients. In addition, the frequency of IP -> IP transition was < 5 for the purulent SSTVI. Therefore, we assumed that the probability of IP -> IP for purulent SSTVI remained the same as that for non-purulent SSTVI.

*Other Transition Probabilities*

For each type of SSTVI, we computed the probability of transitioning from one healthcare setting to another (e.g., from outpatient to inpatient; **Table S1**). For some of the transition probabilities in purulent SSTVI (ED -> SSTVI, ED -> SSTVI Death, IPC -> SSTVI Death, IP -> IP), we were unable to calculate the exact probabilities because the cell sizes were < 5. The aforesaid regulations by the *Institut de la Statistique du Québec* applied here as well. We assumed that the probabilities corresponding to transition in non-purulent SSTVI held true for purulent SSTVI.

*Patient-Directed Discharges*

To account for patients who leave ED or hospital against medical advice (i.e., patient-directed discharges), we assumed that patients transition from ED to the SSTVI health state and from hospital to the SSTVI health state. We assumed that such behaviour reflected patients resorting to self-treatment without having fully recovered from the SSTVI. To capture patient-directed discharges in ED settings, we looked at the records with the first triage time but no time at which the patient is treated by the doctor. To capture this in inpatient settings, we looked at the hospital admission records with the ‘type of destination’ indicating “Departure without authorization” (‘31’).

*Costs Associated with Patient-Directed Discharges*

When patients leave the emergency department or hospital against advice, fewer healthcare resources are spent for those encounters compared to situations where the patient is discharged upon authorization. However, we treated these cases like regular ED visits and hospitalizations. We acknowledge that this approach may underestimate the average costs of ED visits and hospitalizations, as the amount of healthcare resources dedicated to treating the patient may not have been utilized fully in cases of early departures. We took this approach because when we attempted to create a separate health state for patient-directed discharges (PDD), transition from PDD to health service use (e.g., ED and IP) resulted in cell size < 5 (Note: As per regulations dictated by data holders, any frequencies < 5 could not be exported for research use.). In addition, we assumed that there was no spontaneous clearance of SSTVI upon departure from medical institution against medical advice, and we expected these patients to be re-admitted to ED and hospitals due to complications and worsened health outcomes [10]. In our attempt to capture PDD in the model, the harms associated with SSTVI, especially inpatient cases, may have been underestimated. This is because we assumed that when the patients left medical institutions in a self-directed manner, they would transition into the ‘SSTVI’ health state, which had higher health state utility than the hospitalization states. Thus, the average costs and health state utilities presented in the ED and hospitalization health states reflect conservative estimates of the actual costs and QALY at each of these health states. To mitigate potential biases from the underestimation of average costs and harms, we conducted a probabilistic sensitivity analysis, in which we drew random samples of cost and QALY parameters for each health state multiple times from a statistical distribution.

*Transition and Disease Progression*

To calculate the incidence proportion of SSTVI in PWID, we calculated how many PWID there were each week and identified the number of SSTVI episodes at each time point. We then computed how many unique individuals had a medical record of SSTVI in the administrative databases during each week (between January 2009 and March 2019). We calculated the percentage of infected individuals from each week to derive the incidence proportion of SSTVI in any given week. We chose to take the median value of the weekly proportions as the incidence proportion in any given week because given the very low values of incidence proportion (point estimate = 0.001452; 5^th^-95^th^ percentile = 0.00097-0.00296), time trends (e.g., seasonality) were likely to be minor.

To compute the probability of developing SSTVI in any given week, we identified PWID without SSTVI and calculated the number of them for each week. For each week (starting from baseline + 1 week), we calculated the number of incident SSTVI. We divided the number of SSTVI in the (j + 1)-th month by the number of SSTVI-free PWID in the j-th month to obtain the probability of SSTVI.

*Injection Risk Behaviour*

We used the survey response data from the Quebec Provincial Public Health Institute (INSPQ) to parameterize injection risk behaviour, including the frequency of injection in the past month, the number of injections in the past month, and the proportion of PWID who used needles and/or syringes used by someone else [11].

The INSPQ questionnaire on the “number of injections in the past month” included 8 response categories. For the purposes of this model, we grouped them into four categories (1: 1-4 and 5-10; 2: 11-20 and 21-40; 3: 41-60 and 61-100; and 4: 101-200 and 201-9000). In our simulated cohort, we assumed that the patients with a certain level of “injection frequency in the past month” could have only had a certain “number of injections in the past month”. That is, PWID whose injection frequency in the past month is “Not every week” could only fall into group 1 (number of injections in the past month). Similarly, PWID whose injection frequencies were “1-2 days a week”, “3-6 days a week”, and “every day” fell into groups 2, 3, 4, respectively. Within each of the four groups, we simulated the distribution of the number of injections in the past month, so that the original eight categories from the INSPQ questionnaire are reflected in the study population. The number of injections in the past month was set to 0 for PWID who did not inject drugs in the past month.

*Probability of SSTVI*

Since NSP modifies injection risk behaviours, we assumed that the distribution of clinical risk factors for SSTVI (e.g., comorbidities) remained the same across the ‘NSP’ and the ‘No NSP’ groups. Thus, we generated the data for the following demographic and behavioural risk factors: (1) Injection frequency in the past month; (2) Number of injections in the past month; (3) Needle Sharing; (4) Reusing injection equipment; (5) Years of injection drug use; (6) Sex; and (7) Age. We assumed that these seven demographic and behavioural factors at baseline influenced the individual’s probability of SSTVI.

To derive the individual probability of SSTVI, we computed the probability of SSTVI in the general population, including individuals who do not use drugs. We set this value as the probability of SSTVI if the PWID did not inject in the past month, did not share needles, did not reuse injection equipment, had < 5 years of injection drug use, was a female, and belonged to the lowest age group (< 25 years). For PWID with any of the aforementioned behavioural risk factors, this “base” probability was multiplied by the “risk multiplier” (i.e., risk ratios or odds ratios from the literature).

*Risk Multipliers*

We assumed that PWID with more than one behavioural risk factor had a higher risk of SSTVI than those with only one risk factor present, and that the presence of each additional risk factor further increased the likelihood of SSTVI (i.e., multiplicative interaction [12]). However, we do not know the extent of the interaction between risk factors and how they elevate the risk of SSTVI. During the data generation step, we included seven demographic and behavioural risk factors, which were (1) Injection frequency in the past month; (2) Number of injections in the past month; (3) Needle Sharing; (4) Reusing injection equipment; (5) Years of injection drug use; (6) Sex; and (7) Age. Out of the seven risk factors, we took the product of the highest risk ratio and the four lowest risk ratios and multiplied this value by the “base” probability to derive a more conservative estimate of the probability of SSTVI for individuals with the behavioural risk factors.

*Needle and Syringe Program*

The information on the annual cost of operating a NSP in Quebec was not available. Therefore, we used data from Ontario to estimate our operating costs. The total annual cost of NSP in the entire province of Ontario in 2016 was $6.65 million, which corresponded to the annual cost of $279 per person who injects drugs [13]. We assumed that the NSP operation and logistics remained the same during the 5-year study period. Therefore, we applied the Consumer Price Index for ‘Health and Personal Care Products’ for Quebec for 2022 to represent the aforesaid per-person NSP costs over the study period in 2022 Canadian dollars [14].

In addition, the NSP remains open to all PWID who choose to utilize it. To account for the cost of NSP operation, we divided the total cost by 52 to derive the weekly estimate of the cost of NSP per PWID. We added this cost to all individuals in the cohort at each cycle of the follow-up until they reached the death state, in which the NSP costs were no longer incurred.

We assumed that the NSP helps modify people’s injection behaviour, but that it does not influence physician and health system performance. That said, the lack of NSP suggests that more people are sharing and reusing injection equipment, and these are behaviours associated with higher risk of SSTVI. Accordingly, for the scenario without NSP, we set the prevalence of SSTVI to be higher than in the scenario with NSP. However, if the individuals had the same set of injection risk behaviours but were in different NSP scenarios (i.e., some were in the ‘With NSP’ scenario, whereas others were in the ‘No NSP’ scenario), the probability of SSTVI was kept the same regardless of which NSP scenario the individual was observed. In other words, regardless of whether NSP was present or not, the individuals who had the same set of risk factors had the same risk of infection. The main difference between the two NSP scenarios is that the prevalence of SSTVI remained higher in the ‘No NSP’ scenario.

*Cost Elements*

We recognize that in each treatment setting (e.g., outpatient, ED, and inpatient), individuals may incur different cost amounts due to variations in care received. However, in this model, we took the average cost of treating the case at each of the settings for each type of SSTVI to determine the cost of being in a particular health state.

For each unique contact with the healthcare system, we calculated its cost by taking the sum of the cost of the medical visit (e.g., outpatient visit, emergency department visit, or hospitalization) and the cost of prescription medication(s) dispensed at the pharmacy on the date of discharge (for ED visits and hospitalizations) or the date of physician visit (for outpatient cases). To calculate the costs associated with prescription drugs, we included antibiotics, as these are the most commonly prescribed medications to patients with SSTVI. The antibiotics currently available in Canada include trimethoprim-sulfamethoxazole (TMP-SMX), cefalexin, cefazolin, ceftriaxone, clindamycin, cloxacillin, daptomycin, linezolid, vancomycin, amoxicillin, and doxycycline.

Health Canada's DIN corresponding to the above antibiotics are as follows:

- Cefazolin: 02108119, 02108217, 02108135, 02233853, 02233854, 02233855, 02237137, 02237138, 02237140, 02237141, 02297191, 02297205, 02297213, 02308932, 02308959, 02308967, 02318830, 02401029, 02437104, 02437112, 02437120, 02452162, 02465469, 02465477, 02496313, 02524791
- Ceftriaxone: 02250276, 02250284, 02250292, 02250306, 02257866, 02257874, 02287625, 02287633, 02287641, 02287668, 02289679, 02289687, 02289695, 02289709, 02289717, 02292262, 02292270, 02292289, 02292297, 02292815, 02292866, 02292874, 02292882, 02292904, 02325594, 02325608, 02325616, 02325624, 02325632, 02409968, 02465892, 02465906, 02465914, 02465922, 02499711, 02499738
- Clindamycin: 02051826, 02130033, 02130866, 02139286, 02192659, 02230535, 02230540, 02241709, 02241710, 02242409, 02242410, 02245232, 02245233, 02245830, 02245831, 02245832, 02248525, 02248526, 02258331, 02258358, 02266938, 02293382, 02293390, 02294826, 02294834, 02336235, 02336243, 02364719, 02364727, 02385716, 02391643, 02391651, 02400529, 02400537, 02408503, 02408511, 02408538, 02436906, 02436914, 02440091, 02440180, 02462656, 02462664, 02464519, 02468476, 02468484, 02479923, 02479931, 02483734, 02483742, 02483769, 02485109, 02485117, 02485451, 02485478, 02485486, 02493748, 02493756
- Cloxacillin: 00337757, 00337765, 00337773, 01912194, 01912410, 01912429, 01975447, 02030500, 02030519, 02069660, 02069679, 02241195, 02241196, 02367408, 02367416, 02367424, 02400081, 02510731, 02510758
- Daptomycin: 02460009, 02460017, 02462117, 02490463, 02490838, 02494590, 02511738, 02518856, 02526743, 02540266, 02541920
- Linezolid: 02359235, 02402637, 02422689, 02426552, 02429527, 02430827, 02441012, 02457903, 02461897, 02462214, 02469294, 02470551, 02481278, 02486156, 02520354, 02539748
- Vancomycin: 00015423, 00722146, 01990853, 01990861, 01990888, 02015110, 02139243, 02139375, 02139383, 02230191, 02230192, 02241807, 02241820, 02241821, 02342855, 02342863, 02377470, 02377489, 02378337, 02378345, 02394626, 02394634, 02394642, 02394650, 02396386, 02405822, 02405830, 02406497, 02406500, 02406535, 02406543, 02406551, 02406578, 02407744, 02407752, 02407914, 02407922, 02407930, 02407949, 02411032, 02411040, 02420295, 02420309, 02420317, 02420325, 02430185, 02430193, 02435713, 02435721, 02477793, 02477807, 02477815, 02487063, 02487071, 02502593, 02502607, 02531127, 02531135, 02532999, 02533006, 02533014
- Amoxicillin: 00574910, 00632625, 00795925, 02184613, 02229582, 02229583, 02229584, 02230243, 02230244, 02230245, 02230246, 02230615, 02230616, 02230617, 02230618, 02233017, 02237154, 02238171, 02238172, 02240829, 02240830, 02241826, 02241827, 02243224, 02243225, 02262851, 02262878, 02262886, 02262894, 02319594, 02319608, 02345501, 02345528, 02345536, 02345544, 02345552, 02348012, 02348020, 02351153, 02351161, 02351188, 02351196, 02352710, 02352729, 02352737, 02352745, 02352753, 02352761, 02352788, 02388073, 02388081, 02401495, 02401509, 02401533, 02401541, 02401568, 02401576, 02433060, 02433079, 02434709, 02434717, 02447584, 02458586, 02458594, 02470780, 02474751, 02474778, 02477718, 02477726, 02495856, 02495864, 02495910, 02495929, 02495937, 02414648, 02514656, 02514664, 02514672, 02515598, 02515601, 02515628, 02525348, 02525356, 02532042, 02532050, 02535793, 02535815, 02536021, 02536048, 02537451
- Doxycycline: 00725250, 00742562, 02044668, 02044676, 02091232, 02093103, 02140039, 02142058, 02158574, 02199181, 02199203, 02231771, 02289431, 02289458, 02289466, 02289539, 02289547, 02289598, 02347679, 02347687, 02351234, 02341242, 02512645, 02517817, 02528568, 02528940, 02536250, 02543478

To calculate the costs associated with ED visits, we took the unit cost set by the Quebec Ministry of Health and Social Services based on its “Regulation respecting the application of the Hospital Insurance Act” (Chapter A-28, r.1; $1,129.47 per visit in 2023 Canadian dollars) [15], converted it to 2022 Canadian dollars ($1,077.42), and applied it to ED visits that did not involve patient-directed discharges. Since we were unable to capture the cost of diagnostic tests associated with each ED visit in our administrative data, we applied the unit cost ($1,077.42) to ED visits to derive a more conservative estimate of the ED visits. To account for the intensity of ED visits, we assumed that the visits involving patient-directed discharges (i.e., visits with triage time stamp but no records of admission) incurred the cost of triage ($128.15 in 2022 CAD at the JGH). For ED visits involving the use of intensive care unit (ICU), we multiplied the number of days in the ICU by the cost per day in ICU ($11,618.67 per day in 2022 CAD). If the amount of time spent in the ED was less than 24 hours, we considered the length of that visit to have been 1 day. Similarly, if the amount of time spent at in the ED was greater than 24 hours but less than 48 hours, we considered the length of that visit to have been 2 days.

Each hospitalization and day surgery (in hospital settings) had a resource intensity weight, or the *niveau d'intensité relative des ressources utilisées* (NIRRU), associated with it. We obtained the unit cost of 1 NIRRU associated with hospitalization and day surgery from the Quebec Ministry of Health and Social Services and multiplied this value by the NIRRU recorded for each inpatient record in the administrative database (Note: The unit cost of NIRRU for 2017-2018 was not available in public domain. To derive the cost for 2017-2018, we conducted interpolation using the NIRRU costs from 2016-2017 and 2018-2019.). Where the NIRRU was missing or not recorded in the database, we estimated the cost of such hospitalizations by using Canadian Institute for Health Information’s patient cost estimator [16]. We first calculated the cost of one day of hospitalization for the purulent and non-purulent SSTVI in the general population of ages 18-59. For abscess, this corresponded to the Case Mix Group (CMG) code 406 ($2,074.36 per day in 2022 CAD). For cellulitis, this corresponded to the CMG code 405 ($1,743.96 per day in 2022 CAD). For hospitalizations that required surgical interventions and necrotizing fasciitis, we chose the CMG code 392 (“Other skin/subcutaneous tissue intervention”; $2,384.88 per day in 2022 CAD). Then, we multiplied the average cost of 1 day of hospitalization for these conditions by the length of stay to derive the cost of hospitalization in 2022 CAD.

*Statistical Analysis from the Microsimulation Model*

The microsimulation model served as the basis for both economic evaluation and statistical analyses (e.g., survival analysis). This simulation created matrices for costs, QALY, health states (e.g., SSTVI), and health state transitions (e.g., Healthy -> SSTVI), where the number of rows corresponded to the number of patients and the number of columns corresponded to the number of cycles. These matrices informed us about the health state of each patient as well as costs incurred and health state utility at that health state at a specific time point. We used the cost and QALY matrices to conduct economic evaluation and calculate ICER. Relatedly, we used the health state matrix to compute the number of SSTVI mortality in each of the two cohorts and the time point at which each patient reached the “SSTVI mortality” health state. Finally, we used the health state transition matrix to compute the number of contacts with the healthcare system in different settings (e.g., outpatient, emergency department, and inpatient visits) for each of the two cohorts and the time point at which each patient transitioned from the “SSTVI” state to healthcare settings (e.g., outpatient, emergency department, and inpatient visits).

For the survival analysis, we computed the time point during the follow-up at which the individual reached the “SSTVI mortality” state or had contact with the healthcare system. After modifying the format of the dataset to be suitable for survival analysis (i.e., counting process format), we conducted the survival analyses to derive the hazard ratios corresponding to the effectiveness of NSP on SSTVI mortality and on recurrent contacts with the healthcare system.

**Appendix B: Model Calibration**

*Calibration Parameters*

The prior parameter set consisted of 15 risk ratios corresponding to the effectiveness of NSP, injection behaviour risk factors, and demographic characteristics associated with the risk of SSTVI (please refer to Main Manuscript **Table 1** and **Appendix A**, Sections: Probability of SSTVI and Risk Multipliers). These include the following demographic and behavioural risk factors: (1) Injection frequency in the past month; (2) Number of injections in the past month; (3) Needle Sharing; (4) Reusing injection equipment; (5) Years of injection drug use; (6) Sex; and (7) Age. These parameters were selected as calibration parameters because these factors are associated with the risk of acquiring SSTVI. For example, the needle and syringe programs help reduce needle sharing and reuse of injection equipment while contributing to injection cessation [17-22]. At the same time, sharing and reusing of injection equipment, frequency of injections, and years of injection drug use are all known to increase the risk of SSTVI [9, 23-25]. As Quebec-specific information for these risk ratio parameters were not available, we informed our model with parameters from studies conducted in other settings (e.g., United States and United Kingdom). We acknowledge that there may be potential differences in the risk ratios between Quebec and other settings. To account for this limitation, we conducted model calibration to fine-tune these risk ratio estimates, so that these estimates can more accurately reflect the epidemiology of SSTVI and of injection drug use in Quebec.

*Calibration Targets*

We calibrated our model on two calibration targets: other-cause mortality and SSTVI mortality rates, which we defined using the algorithm in Appendix A, Sections “Self-Treatment Mortality”, “SSTVI Mortality”, and “Other-Cause Mortality”. The units corresponding to these two targets were the number of cases per 1,000 population. First, we chose SSTVI mortality rate as one of the calibration targets to ensure that our model accurately estimates the epidemiological burden of SSTVI, given the health service utilization patterns among PWID. Second, we chose other-cause mortality rates as the other calibration target to capture the real-life survival dynamics of the population of PWID and to enhance the model’s validity and credibility.

*Implementation and Results*

We conducted a Bayesian model calibration using the sample importance resampling (SIR) approach [26], where all the risk ratios followed a lognormal distribution. We sampled 2,500 parameter sets from the lognormal distribution, which we then used to run the model 2,500 times (each iteration with 1,000 simulated individuals) and estimate the likelihood in each iteration. Our analysis generated 659unique parameter sets, and we took the median value from these unique sets to derive the “updated” posterior parameter set to approximate the real-life epidemiology and health service use patterns while being less sensitive to outliers and extreme values.

We conducted all subsequent analyses, including base-case analysis, sensitivity analyses, and recurrent-event survival analysis, using the risk ratio values in the “updated” posterior parameter set (Supplementary Table 2). Figures S1 and S2 illustrate the model estimation of mortality rates (and 95% confidence interval [CI]) using the prior and posterior parameter sets in relation to the calibration targets. The target values for OCM and SSTVI mortality derived from the administrative data were 32.76 per 1,000 population (95% CI = 31.83-33.73) and 5.50 per 1,000 population (95% CI = 5.12-5.90), respectively. For OCM, the rates derived from both the prior (rate = 33.85; 95% CI = 33.33-34.38) and the posterior parameter sets (rate = 33.85; 95% CI = 33.33-34.38) were a slight overestimation relative to the calibration target, but the 95% CI from the model estimates overlapped with the 95% CI around the target value. For SSTVI mortality, the rates derived from both the prior (rate = 5.60; 95% CI = 5.39-5.82) and the posterior parameter sets (rate = 5.57; 95% CI = 5.36-5.79) were a slight overestimation relative to the calibration target, but the 95% CI from the model estimates overlapped with the 95% CI around the target value. Calibration results showed that the mortality rates from the posterior parameter set were closer to the target values.

To conduct probabilistic sensitivity analysis, we set the median of the unique parameter sets from the model calibration as the new prior and computed the 95% CI from the 2.5th and 97.5th percentiles of the parameter distributions for each of the calibration parameters. We then sampled from the newly defined distributions (i.e., lognormal distribution for the risk ratios) to perform PSA, ensuring that the variability and uncertainty captured by the SIR process are appropriately incorporated.

*Contact with the Healthcare System*

For individuals with SSTVI, the first contact with the healthcare system took place in either outpatient or emergency department settings. Using the administrative health data, we computed the rate of outpatient visits and ED visits, which were 161.60 and 21.49 per 1,000 person-years, respectively. These rates represent all contacts with the healthcare system in each setting, including repeated claims with the same diagnostic code within a short time frame (e.g., < 7 days), those with the same billing identification number, infections in different parts of the body, and different types of infections. For simplicity, we assumed that only one episode of SSTVI was possible at each cycle, and that there was only one contact with the healthcare system at each cycle for each episode of SSTVI. Due to the model design, our model may underestimate the rate of health service use for each episode and at each cycle. To address these model shortcomings, we calibrated our model so that the cost-utility analysis and survival analysis results reflect clinical and cost-effectiveness when we assume that the rates of health service use from the model were relatively close (+/- 10%) to the rates derived from the administrative data. In our final model, the rates of outpatient visits and ED visits were 147.00 (95% CI = 145.97-148.04) and 21.93 (95% CI = 21.50-22.36) per 1,000 person-years, respectively.

Figure S1. Other-cause mortality as calibration target (posterior versus prior parameter set)


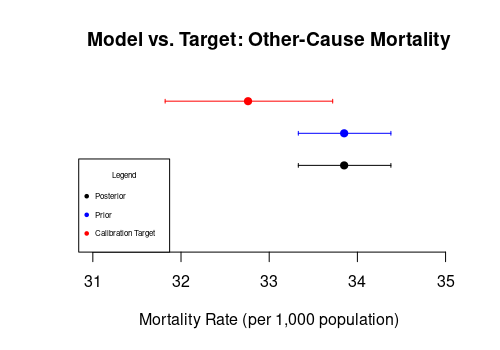


Figure S2. SSTVI mortality as calibration target (posterior versus prior parameter set)

**
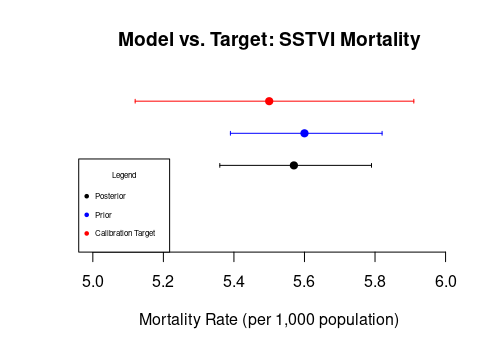
**

**Appendix C: Model Parameters**

Table S1. Derivation formula for transition probabilities

| Parameter | Derivation Formula / Probability |
| --- | --- |
| P (Self-treatment)^1^ | 0.75 |
| P (Health service utilization [HSU])^1^ | 0.25 |
| P (Delays in care)^1, 2^ | 0.65 |
| P (Self-treatment death)^3^ | 0.011012 |
| P (Purulent) | 0.171 |
| P (Non-purulent) | 0.829 |
| P (OP \| purulent) | 0.8565 |
| P (ED \| purulent) | 0.1435 |
| P (OP \| non-purulent) | 0.8720 |
| P (ED \| non-purulent) | 0.1280 |
| P (SSTVI -> SSTVI) | P (Self-treatment) $\times$ P (Delays in care) |
| P (SSTVI -> Self-treatment death) | P (Self-treatment) $\times$ P (Self-treatment death) |
| P (SSTVI -> OP) (purulent) | P (HSU) $\times$ P (Purulent) $\times$ P (OP \| Purulent) |
| P (SSTVI -> ED) (purulent) | P (HSU) $\times$ P (Purulent) $\times$ P (ED \| Purulent) |
| P (SSTVI -> OP) (non-purulent) | P (HSU) $\times$ P (Non-purulent) $\times$ P (OP \| non-purulent) |
| P (SSTVI -> ED) (non-purulent) | P (HSU) $\times$ P (Non-purulent) $\times$ P (ED \| non-purulent) |
| P (OP -> IP) (purulent) | 0.0362 |
| P (OP -> IPC) (purulent) | 0.0223 |
| P (ED -> IP) (purulent) | 0.0333 |
| P (ED -> IPC) (purulent) | 0.0530 |
| P (ED -> SSTVI) (purulent) (PDD) | 0.0025 |
| P (ED -> SSTVI Death) (purulent) (SSTVI death)^4^ | 0.000048 |
| P (IP -> IP) (purulent) (re-admission)^4^ | 0.0124 |
| P (IP -> SSTVI) (purulent) (PDD) | 0.0909 |
| P (IP -> SSTVI Death) (purulent) (SSTVI death)^5^ | 0.000212 |
| P (IPC -> SSTVI) (purulent) (PDD)^4^ | 0.037 |
| P (IPC -> SSTVI Death) (purulent) (SSTVI death)^4^ | 0.000359 |
| P (OP -> IP) (non-purulent) | 0.0411 |
| P (OP -> IPC) (non-purulent) | 0.0042 |
| P (ED -> IP) (non-purulent) | 0.0887 |
| P (ED -> IPC) (non-purulent) | 0.0198 |
| P (ED -> SSTVI) (non-purulent) (PDD) | 0.0025 |
| P (ED -> SSTVI Death) (non-purulent) (SSTVI death)^5^ | 0.000048 |
| P (IP -> IP) (non-purulent) (re-admission) | 0.0124 |
| P (IP -> SSTVI) (non-purulent) (PDD) | 0.0579 |
| P (IP -> SSTVI Death) (non-purulent) (SSTVI death) | 0.000387 |
| P (IPC -> SSTVI) (non-purulent) (PDD) | 0.037 |
| P (IPC -> SSTVI Death) (non-purulent) (SSTVI death)^5^ | 0.000359 |

^1^ These values were based on assumptions. All other transition probabilities were derived from administrative data.

^2^ The probability of delays in care accounted for the likelihood of self-resolution, delays in seeking care from a doctor, not seeking treatment, and applying self-treatment.

^3^ This is the probability of SSTVI mortality following self-treatment when there was no contact with the healthcare system for 15+ days prior to death.

^4^ Due to small cell count (< 5), we assumed that these transition probabilities for purulent SSTVI remained the same as that for non-purulent SSTVI.

^5^ The original values denoted the probability of SSTVI death within 365 days of ED, IP, or IPC (due to zero count cells). To derive the probability of SSTVI death within 7 days of contact with the system, we applied the probability to rate to probability conversion formula.

Table S2. Risk ratios before and after model calibration

| Parameter | Risk Ratios  (95% CI)  Before Calibration | Risk Ratios  (95% CI)  After Calibration | Distribution | Source |
| --- | --- | --- | --- | --- |
| Effectiveness of NSP against sharing needles | 0.42  (0.25-0.72) | 0.417  (0.236-0.705) | Lognormal | [18] |
| Effectiveness of NSP against reusing needles | 0.79  (0.66-0.95) | 0.789  (0.649-0.952) | Lognormal | [18] |
| Sharing needles | 3.31  (2.04-5.37) | 3.286  (2.108-5.216) | Lognormal | [25] |
| Reusing needles | 2.1  (1.2-3.7) | 2.046  (1.207-3.542) | Lognormal | [23] |
| Injection frequency  (< 1 day a week) | REF | REF |  | [23] |
| Injection frequency (1-2 days a week) | 2.1  (1.1-4.0) | 2.070  (1.079-3.789) | Lognormal |  |
| Injection frequency (2-7 days a week) | 3.1  (1.7-5.5) | 3.058  (1.622-5.546) | Lognormal |  |
| Number of injections in the past month (0-10) | REF | REF |  | [23] |
| Number of injections in the past month (11-40) | 2.6  (2.0-3.5) | 2.613  (1.960-3.348) | Lognormal |  |
| Number of injections in the past month (41-100) | 3.7  (2.7-5.2) | 3.694  (2.670-5.146) | Lognormal |  |
| Number of injections in the past month (101-9000) | 3.8  (2.8-5.1) | 3.799  (2.863-5.173) | Lognormal |  |
| Years of injection drug use (0-2 years)^1^ | REF | REF |  | [9] |
| Years of injection drug use (2-5 years) | 2.49  (1.16-5.34) | 2.574  (1.177-5.303) | Lognormal |  |
| Years of injection drug use (5-8 years) | 3.95  (1.73-9.02) | 3.966  (1.764-8.887) | Lognormal |  |
| Years of injection drug use (8+ years) | 4.84  (2.14-10.92) | 4.921  (2.282-10.628) | Lognormal |  |
| Age < 25 | REF | REF |  | Administrative data |
| Age 25-44 | 1.02  (0.98-1.06) | 1.021  (0.984-1.059) | Lognormal |  |
| Age 45+ | 1.16  (1.12-1.21) | 1.163  (1.119-1.207) | Lognormal |  |
| Female | REF | REF |  | Administrative data |
| Male | 1.01  (0.99-1.03) | 1.010  (0.990-1.030) | Lognormal |  |

Abbreviations: CI = Confidence interval; REF = Reference

^1^ Due to the nature of administrative data, there remained uncertainty in capturing the duration of injection drug use among individuals identified as PWID. We assumed that PWID identified in the administrative databases had initiated injection drug use 6 months before being captured in the data. This resulted in small percentage of individuals in the ‘< 1 year of injection drug use’ category. To account for this, we set ‘< 2 years’ of injection drug use as the reference category. Based on patient information from the administrative data, we created the remaining categories of duration of injection drug use as ‘2-5 years’, ‘5-8 years’, and ‘> 8 years’ for the analysis.

REFERENCES

1. Mulliken, J.S. and S.M. Doernberg. *UCSF Medical Center Guideline for the Management of Suspected Skin and Soft Tissue Infections in Adults*. 2019 [cited 2023 October 10, 2023]; Available from: <https://idmp.ucsf.edu/sites/g/files/tkssra4251/f/UCSF%20SSTI%20Guideline%20FINAL_0.pdf>.

2. Stevens, D.L., et al., *Practice guidelines for the diagnosis and management of skin and soft tissue infections: 2014 update by the infectious diseases society of America.* Clin Infect Dis, 2014. **59**(2): p. 147-59.

3. Lewer, D., M. Harris, and V. Hope, *Opiate Injection-Associated Skin, Soft Tissue, and Vascular Infections, England, UK, 1997-2016.* Emerg Infect Dis, 2017. **23**(8): p. 1400-1403.

4. Canadian Institute for Health Information. *CCI: A Guide to Intervention Code Assignment*. 2022 [cited 2023 October 10, 2023]; Available from: <https://www.cihi.ca/sites/default/files/document/CCI-guide-en.pdf>.

5. Canadian Institute for Health Information. *Overview of CCI Sections and Code Ranges*. 2023 [cited 2023 October 12, 2023]; Available from: <https://www.cihi.ca/en/overview-of-cci-sections-and-code-ranges>.

6. Figgatt, M.C., et al., *Treatment experiences for skin and soft tissue infections among participants of syringe service programs in North Carolina.* Harm Reduct J, 2021. **18**(1): p. 80.

7. Ozga, J.E., et al., *A community-based study of abscess self-treatment and barriers to medical care among people who inject drugs in the United States.* Health Soc Care Community, 2022. **30**(5): p. 1798-1808.

8. Monteiro, J., et al., *Self-treatment of skin infections by people who inject drugs.* Drug Alcohol Depend, 2020. **206**: p. 107695.

9. Wright, T., et al., *Prevalence and severity of abscesses and cellulitis, and their associations with other health outcomes, in a community-based study of people who inject drugs in London, UK.* PLoS One, 2020. **15**(7): p. e0235350.

10. Ti, L. and L. Ti, *Leaving the Hospital Against Medical Advice Among People Who Use Illicit Drugs: A Systematic Review.* Am J Public Health, 2015. **105**(12): p. e53-9.

11. Leclerc, P., et al. *Surveillance des maladies infectieuses chez les personnes qui utilisent des drogues par injection*. 2023 [cited 2023 December 28, 2023]; Available from: <https://www.inspq.qc.ca/publications/3362>.

12. VanderWeele, T.J. and M.J. Knol, *A tutorial on interaction.* Epidemiologic methods, 2014. **3**(1): p. 33-72.

13. Optima Decision Science. *Model-based cost-effectiveness and impact assessment of needle-syringe programs in Ontario, Canada from 2006–2015*. 2017 [cited 2023 October 16, 2023]; Available from: <http://optimamodel.com/pubs/Ontario%20NSP%20evaluation%20report.pdf>.

14. Statistics Canada. *Table 18-10-0005-01 Consumer Price Index, annual average, not seasonally adjusted*. 2023 [cited 2023 October 10, 2023]; Available from: <https://www150.statcan.gc.ca/t1/tbl1/en/tv.action?pid=1810000501>.

15. Gouvernement du Québec. *Regulation respecting the application of the Hospital Insurance Act*. January 1, 2024 [cited 2024 May 20, 2024]; Available from: <https://www.legisquebec.gouv.qc.ca/en/document/cr/A-28,%20r.%201>.

16. Canadian Institute for Health Information. *Patient Cost Estimator*. 2023 [cited 2023 September 22, 2023]; Available from: <https://www.cihi.ca/en/patient-cost-estimator>.

17. Werb, D., et al., *Patterns of injection drug use cessation during an expansion of syringe exchange services in a Canadian setting.* Drug Alcohol Depend, 2013. **132**(3): p. 535-40.

18. Bartholomew, T.S., et al., *Reduction in injection risk behaviors after implementation of a syringe services program, Miami, Florida.* J Subst Abuse Treat, 2021. **127**: p. 108344.

19. Marotta, P.L., et al., *Assessing the relationship between syringe exchange, pharmacy, and street sources of accessing syringes and injection drug use behavior in a pooled nationally representative sample of people who inject drugs in the United States from 2002 to 2019.* Harm Reduct J, 2021. **18**(1): p. 115.

20. Fernandes, R.M., et al., *Effectiveness of needle and syringe Programmes in people who inject drugs - An overview of systematic reviews.* BMC Public Health, 2017. **17**(1): p. 309.

21. Sanchez, D.P., et al., *Wounds and Skin and Soft Tissue Infections in People Who Inject Drugs and the Utility of Syringe Service Programs in Their Management.* Adv Wound Care (New Rochelle), 2021. **10**(10): p. 571-582.

22. Thakarar, K., et al., *Injections and infections: understanding syringe service program utilization in a rural state.* Harm Reduct J, 2021. **18**(1): p. 74.

23. Doran, J., et al., *Factors associated with skin and soft tissue infections among people who inject drugs in the United Kingdom: A comparative examination of data from two surveys.* Drug Alcohol Depend, 2020. **213**: p. 108080.

24. Barocas, J.A., et al., *Impact of medications for opioid use disorder among persons hospitalized for drug use-associated skin and soft tissue infections.* Drug Alcohol Depend, 2020. **215**: p. 108207.

25. Jawa, R., et al., *Association of skin infections with sharing of injection drug preparation equipment among people who inject drugs.* Int J Drug Policy, 2021. **94**: p. 103198.

26. Menzies, N.A., et al., *Bayesian Methods for Calibrating Health Policy Models: A Tutorial.* Pharmacoeconomics, 2017. **35**(6): p. 613-624.
